# Surface dose analysis and dosimetric comparison of Halcyon versus Truebeam in breast cancer radiotherapy: An OSL dosimetry study

**DOI:** 10.1002/acm2.70085

**Published:** 2025-04-03

**Authors:** Mustafa Çağlar, Kudret Akçay, Esra Serin, Dursun Eşitmez, Mehmet Sıddık Cebe, Navid Kheradmand, Ömer Yazıcı, Dilek Ünal, Evrim Metcalfe

**Affiliations:** ^1^ Department of Health Physics Graduate School of Health Sciences İstanbul Medipol University İstanbul Turkey; ^2^ Department of Radiation Oncology Bahçelievler Medipol Hospital İstanbul Turkey; ^3^ Program of Radiotherapy Vocational School and Health Service İstanbul Medipol University İstanbul Turkey; ^4^ Program of Radiotherapy Vocational School İstanbul Medipol University İstanbul Turkey; ^5^ Department of Radiation Oncology School of Medicine İstanbul Medipol University İstanbul Turkey; ^6^ Department of Radiation Oncology Medipol Mega University Hospital İstanbul Turkey

**Keywords:** breast cancer, halcyon, O‐Ring linac, OSL, Surface dose, TrueBeam

## Abstract

**Purpose:**

Breast cancer is a neoplastic disease with high prevalence among women. Radiotherapy is one of the principal treatment modalities for this disease, but it poses significant challenges. This study aimed to compare and evaluate the technical and dosimetric performance of conventional C‐arm linac systems and a new design, Halcyon, in the context of breast radiotherapy.

**Methods:**

The study included ten patients who had undergone left breast radiotherapy. Additionally, breast radiotherapy was simulated with an anthropomorphic phantom, and similar planning studies were performed. A total of 40 treatment plans were prepared for ten patients using the field‐in‐field (FinF) and volumetric modulated arc therapy (VMAT) techniques on both TrueBeam and Halcyon systems. Subsequently, treatment plans were created for anthropomorphic phantoms using both techniques on both devices. The dosimetric comparisons were conducted on treatment plans with different treatment techniques on both devices. An anthropomorphic phantom was employed to ascertain the surface dose during treatment, with irradiation conducted in the following with the OSL dosimetry method.

**Results:**

Patient plan comparisons showed no statistically significant differences in planning target volume (PTV) outcomes between techniques and devices. Upon analysis of the organ at risk (OAR), statistically significant differences were identified for FinF in both devices for low‐dose regions. Analysis of the OSL results obtained from phantom irradiations revealed that the Halcyon results were higher than those obtained with the TrueBeam for both techniques. Additionally, a comparison of OSL results with the TPS data revealed discrepancies of up to 18% within the field and up to 22% outside the field. Furthermore, Halcyon demonstrated higher Monitor Unit (MU) values for both techniques, while still maintaining shorter treatment times.

**Conclusion:**

The Halcyon demonstrated comparable technical and dosimetric outcomes to conventional C‐arm linac in breast radiotherapy. Its distinctive design features contribute to the implementation of efficient and secure treatment modalities.

## 1 INTRODUCTION

Breast cancer is a neoplastic disease that arises from the unregulated proliferation of cancer cells within the breast tissue. It is the most prevalent cancer among women, accounting for approximately one‐quarter of all new cases.^1^ Mastectomy and the use of combined therapies such as adjuvant radiotherapy after breast‐conserving surgery are common strategies in the treatment of breast cancer.^2^ Chest wall recurrences, including the scar or dermis layer, are frequently observed following mastectomy. Consequently, the skin is often included as part of the target volume in breast radiotherapy.

It is well known that photons in the mega‐voltage (MV) range used in radiotherapy have a skin‐sparing effect. To increase the surface dose, a water‐equivalent material known as a bolus, typically 0.5 cm or 1 cm thick, is applied to the breast surface.^2^ In breast radiotherapy treatment planning, a technique in which the beams are directed tangentially using appropriate gantry angles and asymmetric jaws has been developed to avoid the overdose of organ at risk (OAR). In conventional tangential breast radiotherapy, beams are placed that provide optimum target volume coverage while protecting the contralateral breast, lung, heart, spinal cord, ipsilateral lung, and contralateral lung.^3^

With the addition of the multi‐leaf collimator (MLC) to the linear accelerator, the field‐in‐field (FinF) technique was introduced to increase dose homogeneity and reduce OAR doses. The use of intensity‐modulated radiation therapy (IMRT) and volumetric modulated arc therapy (VMAT) techniques, which were developed due to technological developments and use the inverse planning method, has increased in breast cancer. Despite the fact that inverse planning offers better dose coverage for tumors and spares organs at risk, the potential for low‐dose spillage in normal tissue, which can elevate the risk of secondary malignancies, remains a concern.^4^

Unlike conventional C‐arm linac, the Halcyon (Varian Medical Systems, Palo Alto, CA) linear accelerator with a novel O‐ring design, was introduced in 2017 to enhance radiotherapy efficiency. The O‐ring geometry allows 4 times faster collimator rotation (2.5 rpm, rotation per minute), and gantry speed (4 rpm) compared to TrueBeam, the C‐arm platform of the same company, without the risk of collision with the patient.^5^ The Halcyon platform has a 6 MV flattening filter free (FFF) photon beam and the maximum dose rate is 800 MU/min. The double‐layer MLC has a stacked stepped design (SX1) with ultra‐low dose leakage (< 0.5%). Halcyon MLC system consists of two layers and the field size formed by these MLCs is 28×28 cm^2^ at the isocenter. The MLC system consists of 114 leaves in total, 58 leaves with the proximal MLC layer and 56 leaves with the distal MLC layer. To reduce linear leaf leakage, the proximal layer offset is 0.5 cm. Leaf speeds are 5.0 cm/s, twice as fast as previous MLCs manufactured.^6^

FFF beams, created by removing the flattening filter, possess lower mean energy than flattened beams due to the elimination of selective attenuation of low‐energy photons, which results in a broader spectral distribution and increased surface dose. With the presence of a flattening filter, the beam hardening effect is greater because the low‐energy photons in the beam are highly attenuated. Significantly less material in the path of the beam in FFF beams reduces this effect and leads to an increase in surface doses.^7^ Additionally, Monte Carlo studies demonstrated that secondary electrons generated in FFF beams contribute more significantly to surface dose compared to flattened beams.^8^, ^9^ Another important aspect that may affect the surface dose in the Halcyon design is the bore cover structure on the beam path. This may act as a beam spoiler by increasing the contribution of scattered low‐energy photons to the surface dose.

In view of these factors, the aim of this study was to compare the treatment plan quality of 10 retrospectively selected breast cancer patients prepared with FinF and VMAT techniques for TrueBeam and Halcyon systems. Additionally, surface dose measurements were conducted to compare in‐field and out‐of‐field doses for breast irradiation on an anthropomorphic phantom in TrueBeam and Halcyon using the optically stimulated luminescence (OSL) dosimetry method.

## 2 MATERIAL AND METHOD

### 2.1 Patient characteristics and planning conditions

In this study, ten female patients who were diagnosed with early‐stage left‐sided breast cancer and treated with TrueBeam at Bahçelievler Medipol Hospital between December 2021 and December 2022 were retrospectively selected and included. Patients were selected based on similar planning target volume (PTV) (mean PTV volume of 1171 cc), compliance with the deep inspiration breath‐hold (DIBH) technique, and the ability to hold their breath.

After the patients and the phantom were positioned in a tilted head‐first supine position using a breast board and a knee wedge, they were sent to the CT simulator (Siemens Biograph mCT‐s40) gantry to obtain images with 2 mm slice thickness. A DIBH scan was conducted in accordance with standard protocol, with the patients being guided to show replicable breathing pattern. This breathing pattern was monitored throughout the procedure using a Varian Real‐time Position Management (RPM) system.

The clinical target volume (CTV) was delineated by a physician in accordance with the RTOG 1005 guideline. The PTV was contoured with a margin of 3 mm to the CTV. Additionally, the PTV was cropped by 2 mm from the lung and 3 mm from the body. The OARs of the left lung, the ipsilateral breast, the heart, the left anterior descending artery (LAD), the esophagus, and the total lung were contoured on the computed tomography (CT) images.

A total dose of 5000 cGy (25 fractions of 200 cGy in 5 weeks) was prescribed, and 95% of PTV received 95% of the prescribed dose in all plans. Care was taken to ensure that the target dose was between 95% and 107% of the prescribed dose. Dose constraints were set based on the RTOG 1005 guidelines (Table 1).^10^

**TABLE 1:** The dose constraints for breast plans were established based on literatures.

Structures	The dose constraints and planning objectives			Reference
PTV	$V_{95\%}\geq 95\%$	$D_{0.03\;\mathrm{cc}}\leq 107\%$		RTOG 1005^10^
Heart	$V_{20\;\mathrm{Gy}}\leq 5\%$	$V_{10\;\mathrm{Gy}}\leq 35\%$	$D_{\mathrm{mean}}\leq 5\;\mathrm{Gy}$	RTOG 1005^10^
LAD	$D_{\max}\leq 20\;\mathrm{Gy}$	$D_{\mathrm{mean}}\leq 8\;\mathrm{Gy}$		Fogliata et al.^11^
Left lung	$V_{20\;\mathrm{Gy}}\leq 20\%$	$V_{10\;\mathrm{Gy}}\leq 40\%$	$V_{5\;\mathrm{Gy}}\leq 55\%$	RTOG 1005^10^
Right lung	$V_{15\;\mathrm{Gy}}\leq 15\%$			RTOG 1005^10^
Contralateral breast	$D_{0.03\;\mathrm{cc}}\leq 5\;\mathrm{Gy}$			RTOG 1005^10^

*Note*: $V_{\mathrm{XX}\%}$ is the volume receiving XX% of the dose; $D_{\mathrm{mean}}$ is the mean dose; $V_{\mathrm{XX}\;\mathrm{Gy}}$ is the volume receiving a dose of XX Gy; $D_{\mathrm{XX}\;\mathrm{cc}}$ is the dose received by XX cc of the volume.

### 2.2 Treatment planning

In this study, treatment plans of retrospectively selected patients and the phantom were prepared for TrueBeam and Halcyon devices using both FinF and VMAT techniques in Eclipse TPS (version 16.1; Varian Medical Systems, Palo Alto, CA, USA).

CT images and radiotherapy structures were imported to Eclipse TPS. Initially, for TrueBeam FinF (TB‐FinF) plans, two tangential fields were applied. No collimator angle was given in the fields set in all plans. Subsequently, FinF fields were created for the hot and cold dose regions. The same isocenter and angles were utilized across the Halcyon and TrueBeam systems, and the dose rate was set to 600 MU/min for the TrueBeam and 800 MU/min for the Halcyon.

In the optimization phase of VMAT treatment plans, the same conditions and priorities were employed in both devices (Table 1). Three short partial arcs irradiating between gantry angles 310°–360° and three short partial arcs irradiating between gantry angles 145°–95° were selected. All plans were conducted for 6 MV FFF beams for Halcyon and 6 MV FF beams for TrueBeam. The Eclipse photon optimization (PO) algorithm was calculated using an Analytic Anisotropic Algorithm (AAA version 16.1). All comparative C‐arm FF linac plans were planned with the FiF technique and reviewed by a physician and were deemed clinically acceptable.^10^

### 2.3 Plan evaluation and plan comparison

The dose statistics of the patient's plan were derived from a dose‐volume histogram (DVH) analysis. In terms of the PTV, conformity index (CI) and homogeneity index (HI) were evaluated with respect to the treatment plans, as outlined in Equations 1 and 2. In order to assess the efficacy of OAR sparing, a series of parameters were evaluated, including heart D_30%_, heart mean dose (D_mean_), ipsilateral lung V_20Gy_, ipsilateral lung V_5Gy_, lungs D_mean_, contralateral breast D_mean_ and LAD D_mean_. Furthermore, the volume of the PTV receiving 105% of the defined dose (V_105%_) was evaluated in order to assess the potential for adverse effects on the breast tissue.

(1) $$\mathrm{CI}\;=\frac{\;\mathrm{PT}{V_{95\%}}^{2}\;}{\mathrm{PTV}\;\times \;TV_{95\%}}\;$$

The term $\mathrm{PT}V_{95\%}$ represents the volume of the PTV that is encompassed by the 95% of the prescribed dose. The PTV itself represents the volume of the PTV, while $TV_{95\%}$ represents the total volume that is encompassed by the prescribed dose. It can be observed that larger CI values indicate a greater degree of conformity with the target. This is in accordance with the findings of Tao Sun et al.^12^

(2) $$\mathrm{HI}=\frac{\;D_{2\%}\;-\;D_{98\%}\;}{D_{p}}\;\;\times \;100\%$$

$D_{2\%}$ indicates dose corresponding to 2% of the volume for the maximum dose, $D_{98\%}$ indicates dose for 98% of the volume for the minimum dose, and $D_{p}$ indicates the prescribed dose. A decrease in HI values suggests greater uniformity of the target.^13^

The number of MU was analyzed for each plan. The delivery time was recorded in seconds from starting with the first field irradiation to the final of last field irradiation during OSL irradiation both techniques for each device, the time required for positioning was excluded from the delivery time.

### 2.4 OSL calibration and phantom measurements

**FIGURE 1:**
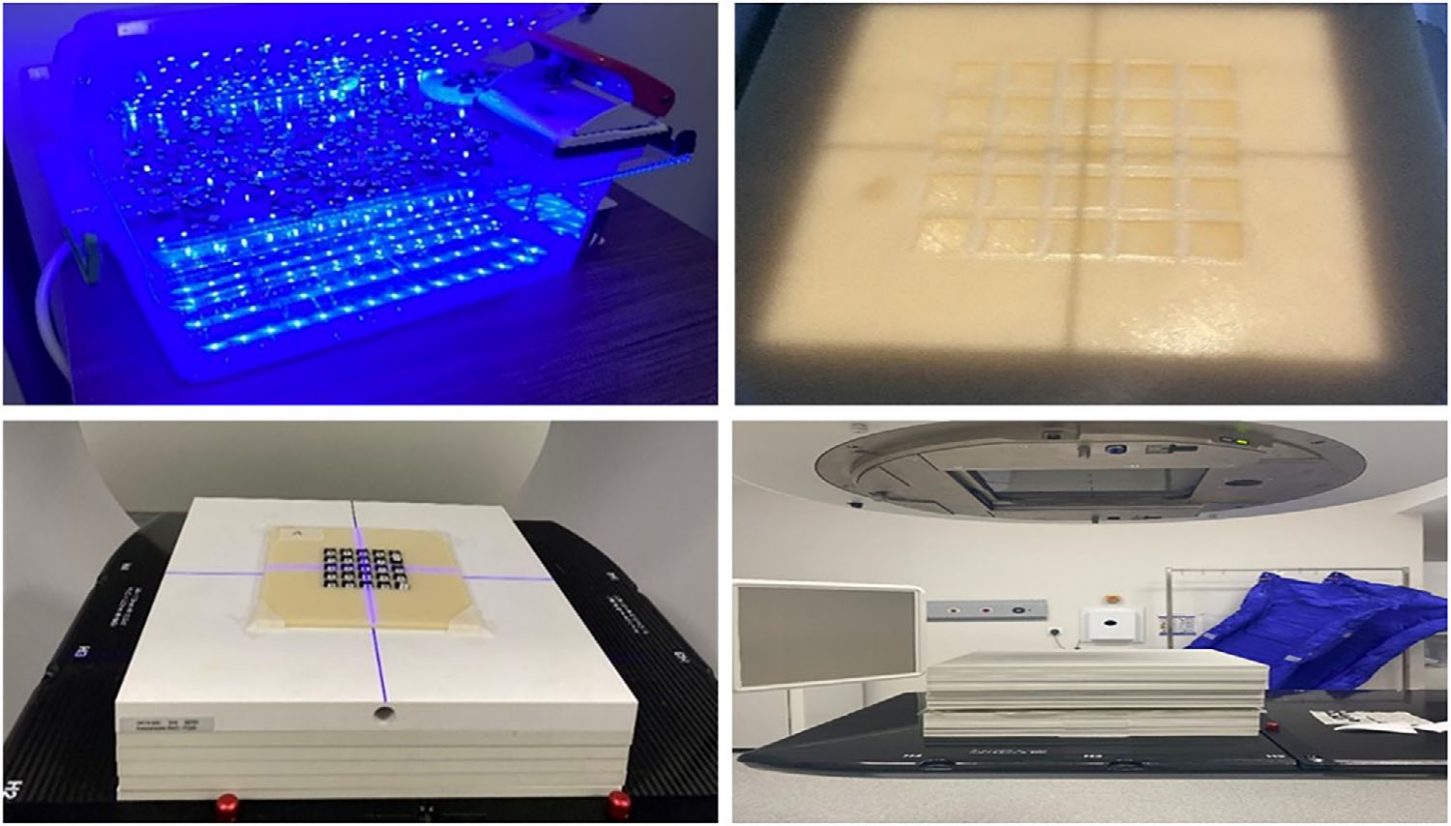
Blue light box used for OSL zeroing (top left), 3D printed OSL holder system (top right), setup preparation (bottom left), and irradiation setup (bottom right).

In order to select the optimal OSL to be utilized in the study, the 112 OSL chips we possessed were initially kept under blue light for twelve hours and zeroed (Figure 1). Subsequently, each OSL chip was checked by reading with an In Light micro Star OSL reader system. In order to calibrate the OSL chips, a series of output and mechanical controls of linac were performed.

After the OSLs were placed in a 3D printed OSL holder system (Figure 1), designed to accommodate 25 nanoDots, the holder cross was exactly matched with the cross of the radiation beam, a 1 cm bolus and 9 solid water phantoms (each with a thickness of 1 cm) were placed on it, and the setup was prepared by setting the source skin distance (SSD) to 100 cm in a 10 × 10 cm^2^ field size. The irradiations were performed three times by giving 100 MU for both devices (Figure 1).

**FIGURE 2:**
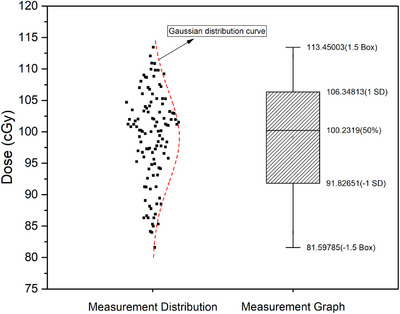
Using the OSL holder, a total of 112 OSL irradiations were performed in 5 sets. The ratio of reading results to PDD_10cm_ was calculated.

A total of 112 OSL dosimeters were irradiated in five sets using an OSL holder. After waiting 12 hours post‐irradiation, 75 OSLs (within 7%) with the closest response to 100 cGy were selected based on reading results and the percentage depth dose at 10 cm depth (PDD_10cm_) factor (Figure 2). The PDD_10cm_ factor was obtained from water phantom data for both TrueBeam and Halcyon systems and was applied to normalize the OSL readings. These 75 OSLs were then exposed to blue light for 12 hours to reset the signal before reuse. Subsequently, three sets of irradiations (100, 200, and 300 MU) were performed for each device, and linearity curves were generated.

Superficial dose measurements were conducted on a phantom that had been treated with FinF and VMAT on Halcyon and TrueBeam. A total of 36 measurement points were identified, including five points on the irradiated breast, five points on the contralateral breast, three points each on the left and right breast, two points each under the left and right breast, and 10 points in the midline region. The measurements were conducted using the Landauer nanoDot OSL dosimetry system.

The Landauer nanodots are composed of carbon‐doped aluminum oxide (Al_2_O_3_:C) disks measuring 7 mm in diameter and 0.2 mm in thickness, which are enclosed in a light‐tight plastic case measuring 1cm×1cm×2mm. The intrinsic buildup of this case has a density of 0.04 g/cm^2^.^14^ The OSL batch was calibrated in accordance with the vendor‐recommended procedure.^15^ The calibration was then validated to an accuracy of 1.5%, which is within the accuracy of 7% claimed by the vendor.

### 2.5 Statistical analysis

All parameters were subjected to analysis using the SPSS tool using the paired t‐test method and a *p*‐value of less than 0.05 was deemed statistically significant. One‐way analysis of variance (ANOVA) was employed for parametric data in comparisons between different techniques used in the study. The homogeneity of variances was evaluated according to the Levene's test. When the *p*‐value was less than 0.05, the variances were deemed to be inhomogeneous. Conversely, when the *p*‐value was greater than 0.05, the variances were considered homogeneous. Consequently, post‐hoc tests were employed for the purpose of making comparisons. The non‐parametric data were analyzed using the Mann–Whitney *U* test.

## 3 RESULTS

### 3.1 TPS dose calculation comparison

Table 2 summarizes the results of the plan quality evaluation index for the comparison between TrueBeam and Halcyon for both FinF and VMAT techniques. For PTV, there was no significant difference in terms of D_mean_, D_2%_, D_98%_, V_107%_, V_110%_, and CI between the TB‐FinF and Truebeam VMAT (TB‐VMAT) techniques, and the Halcyon FinF (HC‐FinF) and Halcyon VMAT (HC‐VMAT) techniques for the target coverage. Concurrently, there was no discernible distinction between the techniques employed on the TrueBeam and Halcyon (*p* > 0.05). Nevertheless, a notable disparity was observed in the HI between TB‐VMAT and HC‐FinF (*p* < 0.05) (Table 2).

**TABLE 2:** Dosimetric parameters of PTV for FinF and VMAT techniques both TrueBeam and Halcyon.

PTV	TB‐FinF	TB‐VMAT	HC‐FinF	HC‐VMAT	*p*‐value
D_mean_ (cGy)	5034.9 ± 31.1	5040.3 ± 59.9	5098.8 ± 70.2	5114.1 ± 97.4	—
D_2%_ (cGy)	5271.4 ± 51.2	5222.8 ± 102.5	5363.4 ± 130.2	5370.6 ± 173.5	—
D_98%_ (cGy)	4573.2 ± 182.4	4699.2 ± 101.7	4612.4 ± 41.9	4675.8 ± 120	—
V_107%_ (%)	0.4 ± 0.6	0.8 ± 1.5	10.8 ± 14.8	11.4 ± 19	—
V_110%_ (%)	0.0 ± 0.0	1.1 ± 2.1	2.2 ± 4.2	2.6 ± 4.6	—
CI	0.948 ± 0.04	0.950 ± 0.04	0.950 ± 0.04	0.947 ± 0.04	—
HI (%)	13.92 ± 2.62	10.47 ± 0.02	15.42 ± 1.77	13.96 ± 1.07	^d^
MU	256.8 ± 46.5	383.6 ± 99.9	326.4 ± 103.9	442.6 ± 100.2	^a^, ^b^, ^c^, ^e^
DeliveryTime (s)	42.4 ± 3.1	92.6 ± 9.3	39.6 ± 4.4	52.8 ± 7.6	^a^, ^c^, ^d^, ^e^

Abbreviations: FinF, field‐in‐field; HC, Halcyon; PTV, planning target volume; TB, TrueBeam; VMAT, volumetric modulated arc therapy.

^a^ TB‐FinF versus TB‐VMAT.

^b^ TB‐FinF versus HC‐FinF.

^c^ TB‐FinF versus HC‐VMAT.

^d^ TB‐VMAT versus HC‐FinF.

^e^ TB‐VMAT versus HC‐VMAT.

^f^ HC‐FinF versus HC‐VMAT.

Table 3 presents the dosimetric parameters of OARs and statistical details pertaining to the techniques and machines employed. The application of FinF techniques (TB‐FinF, HC‐FinF) in both machines resulted in a notable reduction in the D_5%_, D_15%,_ and D_mean_ values of the heart in comparison to the VMAT technique (TB‐VMAT, HC‐VMAT) (*p* < 0.05). No significant difference was observed in the heart for the plans of different treatment devices using the same techniques.

**TABLE 3:** Dosimetric parameters of OARs for FinF and VMAT plans on the TrueBeam and Halcyon.

OAR	TB‐FinF	TB‐VMAT	HC‐FinF	HC‐VMAT	*p‐value*
*Heart*					
D_5%_ (cGy)	511.1 ± 112.3	1684.1 ± 565.2	865.6 ± 792.2	1932.2 ± 433.3	^a^, ^c^, ^d^, ^f^
D_15%_ (cGy)	262.1 ± 34.4	914.1 ± 314.0	499.4 ± 422.1	1154.4 ± 335.4	^a^, ^c^, ^d^, ^f^
Dmean (cGy)	181.3 ± 52.3	504.3 ± 166.1	292.3 ± 184.2	636.1 ± 266.6	^a^, ^c^, ^d^, ^f^
Dmax (cGy)	3723.7 ± 1233.5	3834.8 ± 686.4	3964.0 ± 904.5	4082.9 ± 618.4	–
*Ipsilateral lung*					
V5% (%)	25.4 ± 11,7	53.3 ± 18,1	34.9 ± 22.4	59.6 ± 23.9	^a^, ^c^, ^f^
V20% (%)	11.4 ± 6,1	16.4 ± 5,6	14.2 ± 7.2	20.2 ± 9.6	^a^, ^c^, ^f^
*Lungs*					
Dmean (cGy)	327 ± 147.1	537.5 ± 201.7	415.2 ± 185.3	599.1 ± 353.6	–
*Contralateral breast*					
Dmean (cGy)	11.4 ± 9.2	146.4 ± 163.5	19.4 ± 15.5	163.9 ± 176.9	^a^, ^c^, ^d^, ^f^
Dmax (cGy)	983.2 ± 1412.6	1024.1 ± 625.3	996.2 ± 1556.1	1023.3 ± 652.9	–
*LAD*					
Dmean (cGy)	486.2 ± 61.3	1194.8 ± 392.8	755.3 ± 633.8	1483.5 ± 235.1	^a^, ^c^, ^f^
Dmax (cGy)	1249.2 ± 151.3	2603.8 ± 876.6	1549 ± 1242.1	3332.9 ± 921.4	^a^, ^c^, ^f^
V30% (%)	0 ± 0.1	3.5 ± 5.8	5.6 ± 11.3	6.8 ± 6.7	^a^, ^c^

Abbreviations: OAR, organ at risk; VMAT, volumetric modulated arc therapy; TB‐FinF, TrueBeam‐field‐in‐field.

^a^ TB‐FinF versus TB‐VMAT.

^b^ TB‐FinF versus HC‐FinF.

^c^ TB‐FinF versus HC‐VMAT.

^d^ TB‐VMAT versus HC‐FinF.

^e^ TB‐VMAT versus HC‐VMAT.

^f^ HC‐FinF versus HC‐VMAT.

The study compared the V_5%_ and V_20%_ values of the ipsilateral lung among TB‐FinF, TB‐VMAT, HC‐FinF, and HC‐VMAT plans. Results indicated that there was no significant difference between TrueBeam and Halcyon when using the same techniques. However, the FinF plans demonstrated a significant reduction in V_5%_ and V_20%_ values of the ipsilateral lung compared to the VMAT plans (*p* < 0.05). No significant differences were observed in the D_mean_ to the lungs between the different techniques.

Moreover, the mean doses to the contralateral breast were significantly reduced in the TB‐FinF and HC‐FinF plans when compared to the TB‐VMAT and HC‐VMAT plans (*p* < 0.05). Yet, there were no significant differences in the D_max_ to the contralateral breast. Additionally, in the FinF plans, the doses to the left anterior descending artery (LAD), including V_30%_, D_mean_, and D_max_, were significantly lower compared to the VMAT plans for both TrueBeam and Halcyon (*p* < 0.05). These findings highlight the advantage of FinF plans in minimizing radiation exposure to critical structures while maintaining effective treatment coverage. In addition, the Halcyon device has been shown to deliver higher MU values for both techniques (*p* < 0.05) while maintaining shorter treatment times due to Halcyon's faster gantry speed, MLC speed, and higher dose rate (*p* < 0.05).

### 3.2 OSL measurements comparison

The PDD_10cm_ ratio of the reading results was then calculated. The results were subjected to statistical analysis, which revealed that the histogram is consistent with a Gaussian distribution (skewness test value of −0.422 and kurtosis test value of −0.445). Figure 2 illustrates that the maximum value of the readings was 113.45 cGy, while the minimum value was 81.59 cGy. Furthermore, there are 112 measurements with a mean value of 99.09 ± 7.261 (Figure 2). The 75 OSLs within ± 1 standard deviation (SD) were selected for the subsequent step.

**FIGURE 3:**
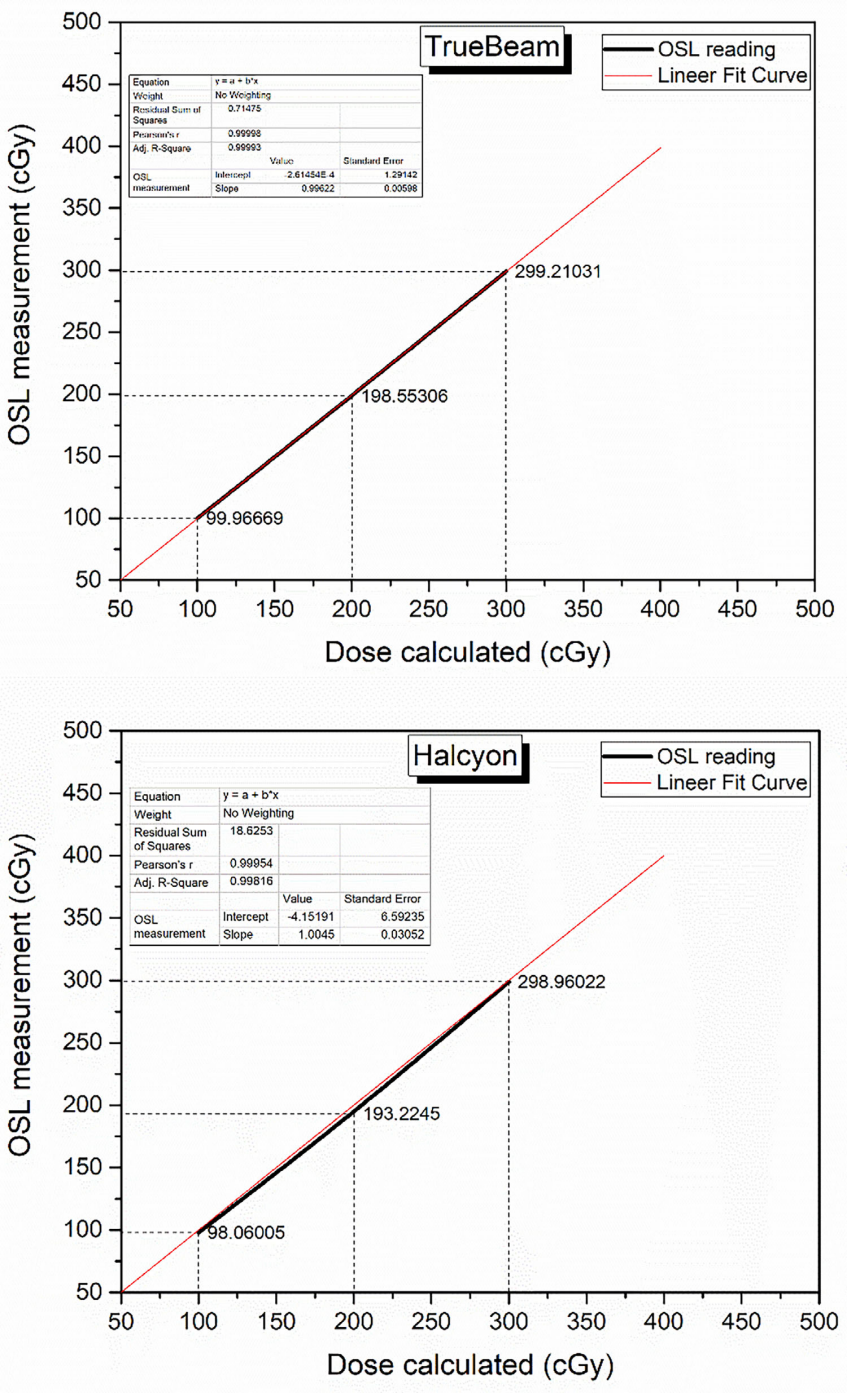
Linearity curves of OSL results obtained with TrueBeam irradiation (Top) and Halcyon irradiation (Bottom).

The linearity graphs obtained by performing the zeroing process again for 75 selected OSLs and irradiating three times for 100 MU, 200 MU, and 300 MU in both devices under the same setup conditions are presented in Figure 3. Consequently, linear fit curves were generated for the energies in both devices (6 MV FF and 6 MV FFF) and Pearson's *r* values were calculated as 0.99998 and 0.99954 for TrueBeam and Halcyon, respectively. It was determined that they exhibited good linearity in the relevant dose range (Figure 3).

The doses at a total of 36 measurement points, determined in nine different regions (left breast, right breast, upper left breast, upper right breast, lower left breast, lower right breast, midline, left lens, and right lens), were read and recorded via TPS for TB‐FinF, TB‐VMAT, HC‐FinF, HC‐VMAT plans. Subsequently, OSL readings were conducted on the phantom irradiations for the TB‐FinF, TB‐VMAT, HC‐FinF, and HC‐VMAT plans. The results of both TPS readings and OSL measurements were averaged by region and are presented in Table 4.

**TABLE 4:** Surface dose results with TPS and OSL for TrueBeam FinF, TrueBeam VMAT, Halcyon FinF, and Halcyon VMAT in different regions. The results show the average scores for each region, ranging from 4 to 6 points.

	TB‐FinF		TB‐VMAT		HC‐FinF		HC‐VMAT	
Region	TPS (cGy)	OSL (cGy)	TPS (cGy)	OSL (cGy)	TPS (cGy)	OSL (cGy)	TPS (cGy)	OSL (cGy)
Left breast	112.21 ± 13.3	125.40 ± 12.58	84.32 ± 4.5	125.40 ± 12.58	112.54 ± 57.4	125.40 ± 12.58	101.52 ± 11.2	125.40 ± 12.6
Right breast	24.22 ± 24.3	26.71 ± 40.23	22.81 ± 10.1	26.71 ± 40.23	27.21 ± 36.6	26.71 ± 40.23	18.35 ± 12.91	26.71 ± 40.23
Left breast top	4.63 ± 12.6	7.18 ± 0.54	3.91 ± 1.4	7.18 ± 0.54	5.12 ± 2.5	7.18 ± 0.54	4.32 ± 2.6	7.18 ± 0.54
Right breast top	3.67 ± 0.6	2.80 ± 0.54	6.42 ± 4.6	2.80 ± 0.54	3.32 ± 0.5	2.80 ± 0.54	5.58 ± 4.3	2.80 ± 0.54
Left breast bottom	8.73 ± 4.3	5.66 ± 0.62	15.11 ± 0.2	5.66 ± 0.45	9.42 ± 2.6	5.66 ± 0.43	7.90 ± 1.4	5.66 ± 0.32
Right breast bottom	4.32 ± 0.8	3.23 ± 0.52	5.54 ± 1.7	3.23 ± 0.45	3.14 ± 0.6	3.23 ± 0.46	4.40 ± 1.2	3.23 ± 0.35
Midline	18.42 ± 38.2	15.29 ± 16.64	36.32 ± 33.4	15.29 ± 16.64	19.86 ± 20.0	15.29 ± 16.64	38.47 ± 32.7	15.29 ± 16.64
Left lens	–	1.30 ± 0.13	–	1.30 ± 0.13	0.21 ± 0.2	1.30 ± 0.13	0.25 ± 0.1	1.30 ± 0.13
Right lens	–	0.96 ± 0.02	–	0.96 ± 0.02	0.11 ± 0.4	0.96 ± 0.02	0.25 ± 0.0	0.96 ± 0.02

Abbreviations: FinF, field‐in‐field; HC, Halcyon; PTV, planning target volume; TB, TrueBeam; VMAT, volumetric modulated arc therapy.

For the left breast, the TPS readings and OSL measurements on the phantom were analyzed. It was observed that the average of the five different points on the left breast was highest in HC‐FinF (114.7 cGy, 138.6 cGy, respectively) and lowest in TB‐VMAT (84.28 cGy, 71.57 cGy, respectively). The greatest deviation between TPS and OSL results was 18%. Upon examination of the out‐of‐field regions, it was found that the TPS results were below 30 cGy for all plans in the right breast. The maximum discrepancy between TPS and OSL measurement results was 22%.

The TPS readings and OSL results for the upper left breast and lower left breast, upper right breast, and lower right breast were below 15 cGy. Upon examination of the midline measurements, it was observed that the highest readings were observed in VMAT techniques for both devices, with OSL readings also demonstrating compatibility with the results. While no dose was calculated in the TPS data of the lenses, which are the critical structures located farthest from the irradiation area, values ​​below 1.5 cGy were obtained in the OSL readings as a result of irradiations.

## 4 DISCUSSION

The objective of this study was to evaluate the quality of treatment plans using different techniques (3DCRT and VMAT) in the treatment of early‐stage left‐sided breast cancer. The plan quality was assessed in terms of target coverage and sparing of OAR, using two different treatment machines (TrueBeam and Halcyon). At the same time, we studied the surface dose on the different points of the phantom using OSL dosimeters both TrueBeam and Halcyon systems.

Our findings indicate that the O‐ring linac did not result in a significant improvement in target coverage compared to the C‐arm. When the techniques are evaluated in terms of HI, it is known that FinF techniques show a lower homogeneity than VMAT techniques. Moreover, HI is associated with numerous mechanical and dosimetric parameters, including radiation energy, MLC structure, MLC speed, dose rate, and gantry rotation.^4^ Statistical analysis has demonstrated that HI is less favorable than HC‐FinF in TB‐VMAT based breast radiotherapy plans. Nevertheless, this difference is not clinically significant. The high breast size also presents a challenge in evaluating the HI parameter. Eunbin Ju et al. compared Halcyon with VitalBeam, a C‐arm linac, in terms of dosimetric parameters for breast irradiation. The results showed that there was no statistical difference in CI and HI for both devices in terms of PTV. Nevertheless, no comparison was made between different techniques in this study.^16^ In this study, the efficacy of two distinct techniques in both devices was evaluated by comparing the resulting treatment plans.

The present study compared the results of OARs sparing data for the heart, lung, opposite breast, and LAD for left breast cancer radiotherapy using FinF and VMAT techniques for TrueBeam and Halcyon systems. For the heart, a significant reduction in D_5%_, D_15%_, and D_mean_ was observed when FinF was used in comparison to VMAT (*p* < 0.05). These findings are consistent with those reported in other VMAT studies of left breast cancer radiotherapy using conventional C‐arm Linac.^17^, ^18^, ^19^, ^20^ The aforementioned discrepancy was also observed in the Halcyon system. TB‐FinF exhibited a notable decline in comparison to HC‐VMAT, while HC‐FinF demonstrated a significant reduction in comparison to TB‐VMAT (*p* < 0.05). No discernible distinction was identified between the techniques and devices with respect to heart Dmax (*p* > 0.05). This can be attributed to the fact that the planner employs maximum dose limitations during the planning and optimization stages.

In addition to cardiac structures, TB‐FinF and HC‐FinF demonstrated superior lung‐sparing efficacy compared to TB‐VMAT and HC‐VMAT, as evidenced by a reduction in V_5%_ in lungs and V5% in the ipsilateral lung using the tangential technique in left breast irradiation. This could potentially reduce the incidence of radiation pneumonitis.^21^, ^22^, ^23^ Conversely, there were no discernible differences in the total lung mean dose between the techniques in the two treatment systems. In a study by Morris et al., the dosimetric differences between Halcyon plans and TrueBeam plans were compared. The results demonstrated that there was no significant difference in ipsilateral lung doses in breast plans for both devices.^24^

The results demonstrated that target coverage, OARs sparing for heart, lungs, contralateral breast, and LAD doses, and machine efficiencies of FinF and VMAT techniques using TrueBeam and Halcyon devices for left‐side breast cancer were comparable in terms of MU and treatment time. When MU values were analyzed, VMAT plans exhibited significantly higher MU values due to increased modulation, whereas FinF plans require lower MU since they deliver a more uniform fluence distribution with fewer segmental variations. Consistently, our study showed that the MU values in VMAT plans were statistically higher than those in FinF plans (*p* < 0.05). Furthermore, it was observed that the MU values in both HC‐FinF and HC‐VMAT techniques were statistically higher than TB‐FinF and TB‐VMAT (*p* < 0.05). This difference is primarily attributed to the absence of a jaw system in the HC device and the utilization of a dual‐layer MLC system.

Despite the higher MU values, this does not result in longer treatment delivery times compared to TrueBeam plans. The Halcyon ring gantry is capable of rotating at a faster rate than that of a conventional TrueBeam, with a maximum speed of four rpm, compared to 1 rpm. Additionally, Halcyon delivers a higher dose rate (800 MU/min) compared to the 6 MV flattened beam on TrueBeam (600 MU/min), further compensating for the increased MU values. In their study, Morris et al. calculated the average delivery times with Halcyon and TrueBeam devices for 10 patients, finding them to be 62.4 s and 57.9 s, respectively.^24^

The results of the study demonstrated a notable elevation in the superficial dose of whole breast irradiation with the Halcyon device in comparison to the TrueBeam 6 MV FF, with a range of 1%–8%. O Gardy et al. compared Halcyon 6 MV FFF and conventional C‐arm 6 MV FF superficial doses in both in vivo and phantom measurements. They showed an average increase of around 10% with Halcyon compared to conventional c‐arm linacs.^2^ Soleymanifard et al. performed a similar study and showed an increase of about 10% with Halcyon compared to C‐arm in surface dose measurements made with thermoluminescence dosimetry.^25^

Upon analysis of surface doses on the left breast, it was determined that the doses were higher in the plans performed with Halcyon than TrueBeam for the same techniques. The primary reason for this increase can be attributed to dosimetric and geometric discrepancies. The presence of a flattening filter in 6 MV FF beams significantly reduces the proportion of low‐energy x‐rays, as the filter preferentially attenuates these photons, leading to a harder beam and a more uniform dose distribution. In contrast, the absence of a flattening filter in 6 MV FFF beams allows a greater number of low‐energy x‐rays to reach the patient's surface, resulting in an increased surface dose and a softer beam spectrum.

Furthermore, an analysis of the geometrical structure of the Halcyon system indicates that the bore cover system may introduce a spoiler effect. This effect promotes the production of additional secondary electrons, which subsequently contribute to an elevated surface dose by increasing electron contamination at the patient's surface. Nevertheless, studies indicate that its impact is approximately 1%, with the primary influence emanating from the elevated fluence of contamination electrons in the absence of the flattening filter.^2^ The lack of a significant difference in device performance across different techniques indicates that device efficiencies are comparable.

It was observed that the relative increase in superficial dose predicted by AAA for Halcyon versus C‐arm FF linac was smaller compared with that observed in the in vivo and phantom measurements. This is most likely a reflection of the limited accuracy of the surface dose modeling in the current AAA algorithm. Caution is advised when evaluating superficial dose for Halcyon plans as it might underestimate the actual dose received by superficial structures.

## 5 CONCLUSIONS

In this study, the performance of the Halcyon system, which has a new design and uses FFF beams in whole‐breast radiotherapy, was compared with conventional C‐arm linac systems. No statistically significant dosimetric differences were observed between treatment plans prepared with 3DCRT and VMAT techniques, especially between treatment plans prepared for different devices. The workflow described above for breast irradiation with Halcyon has been developed aiming to ensure patient safety, maintain treatment precision, and enable dosimetric comparison with conventional C‐arm systems. The workflow is analogous to that of C‐arm linac and comprises the same preparatory steps for treatment, thus eliminating the need for additional training for personnel. Dosimetric discrepancies that may emerge from the novel design were examined with both TPS data and phantom measurements for breast irradiation, and it was determined that there were no clinically significant differences. Nevertheless, further investigation in vivo for diverse scenarios is warranted to provide guidance on clinical safety.

## AUTHOR CONTRIBUTIONS

Mustafa Çağlar: Supervision and project administration. Drafting the work, figure/table preparation, provided oversight, guidance, and final review and approval of the manuscript. Kudret Akçay: Radiotherapy planning, conceptualization, study design, data collection, OSL measurement procedure. Dursun Eşitmez: Assisted in treatment planning procedures and verification of dosimetric data. Esra Serin: Contributed to patient selection criteria and radiotherapy planning. Mehmet Sıddık Cebe: Review, and manuscript language editing. Contributed to drafting and critical revision of the manuscript for important intellectual content. Navid Kheradmand: Review, manuscript language editing, and figure/table preparation. Ömer Yazıcı: Dosimetric equipment support, verification of dosimetric data, review the treatment planning. Dilek Ünal: Dosimetric equipment support, verification of dosimetric data, review the treatment planning. Evrim Metcalfe: Contributed to patient selection criteria and approved radiotherapy planning.

## CONFLICT OF INTEREST STATEMENT

The authors declare no conflicts of interest.
